# (4R*,5R*)-2-(4-Meth­oxy­phen­yl)-1,3-dioxolane-4,5-dicarboxamide

**DOI:** 10.1107/S1600536812002401

**Published:** 2012-02-04

**Authors:** Chun-Lei Lv, Jian-Hui Chen, Yu-Zhe Zhang, Ding-Qiang Lu, Ping-Kai OuYang

**Affiliations:** a School of Pharmaceutical Science, Nanjing University of Technolgy, Xinmofan Road No. 5 Nanjing, Nanjing 210009, People’s Republic of China; b Xinchang Pharmaceutical Factory, Zhejiang Medicine Co. Ltd, Xinchang 312500, People’s Republic of China; c College of Materials Science and, Engineering, Nanjing University of Technology, Xinmofan Road No. 5 Nanjing, Nanjing 210009, People’s Republic of China

## Abstract

In the title compound, C_12_H_14_N_2_O_5_, the five-membered 1,3-dioxolane ring has a twisted conformation. In the crystal, N—H⋯O and C—H⋯O hydrogen bonds link the mol­ecules into a two-dimensional network lying parallel to the *ab* plane. There are also C—H⋯π inter­actions present in the crystal structure.

## Related literature

For the importantce of (2*S*,3S)-diethyl-2,3-*O*-alkyl­tartrate analogues in the synthesis of platinum complexes with anti­tumor activity, see: Kim *et al.* (1994 ▶), and for their importance as inter­mediates in organic synthesis, see: Pandey *et al.* (1997 ▶). For the synthesis of the title compound, see: Ates-Alagoz & Buyukbingol (2001 ▶). For standard bond lengths, see: Allen *et al.* (1987 ▶).
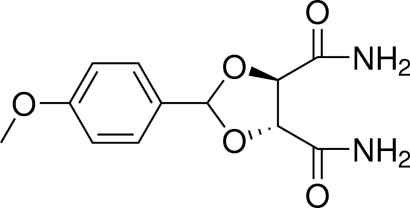


## Experimental

### 

#### Crystal data

C_12_H_14_N_2_O_5_
*M* *_r_* = 266.25
Orthorhombic, 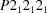

*a* = 6.9620 (14) Å
*b* = 10.727 (2) Å
*c* = 16.932 (3) Å
*V* = 1264.5 (4) Å^3^
*Z* = 4
Mo *K*α radiation
μ = 0.11 mm^−1^
*T* = 293 K
0.20 × 0.20 × 0.10 mm

#### Data collection

Enraf–Nonius CAD-4 diffractometer
Absorption correction: ψ scan (North *et al.*, 1968 ▶) *T*_min_ = 0.978, *T*_max_ = 0.989
2615 measured reflections
2297 independent reflections
1939 reflections with *I* > 2σ(*I*)
*R*_int_ = 0.035
3 standard reflections every 200 reflections intensity decay: 1%

#### Refinement

*R*[*F* ^2^ > 2σ(*F* ^2^)] = 0.041
*wR*(*F* ^2^) = 0.135
*S* = 1.00
2297 reflections
173 parameters
H-atom parameters constrained
Δρ_max_ = 0.16 e Å^−3^
Δρ_min_ = −0.16 e Å^−3^

### 

Data collection: *CAD-4 Software* (Enraf–Nonius, 1989 ▶); cell refinement: *CAD-4 Software*; data reduction: *XCAD4* (Harms & Wocadlo, 1995 ▶); program(s) used to solve structure: *SHELXS97* (Sheldrick, 2008 ▶); program(s) used to refine structure: *SHELXL97* (Sheldrick, 2008 ▶); molecular graphics: *PLATON* (Spek, 2009 ▶); software used to prepare material for publication: *SHELXL97*.

## Supplementary Material

Crystal structure: contains datablock(s) global, I. DOI: 10.1107/S1600536812002401/su2367sup1.cif

Structure factors: contains datablock(s) I. DOI: 10.1107/S1600536812002401/su2367Isup2.hkl

Supplementary material file. DOI: 10.1107/S1600536812002401/su2367Isup3.cml

Additional supplementary materials:  crystallographic information; 3D view; checkCIF report

## Figures and Tables

**Table 1:** Hydrogen-bond geometry (Å, °) *Cg*2 is the centroid of the C2–C7 ring.

*D*—H⋯*A*	*D*—H	H⋯*A*	*D*⋯*A*	*D*—H⋯*A*
N1—H1*A*⋯O1^i^	0.86	2.21	3.063 (3)	174
N1—H1*B*⋯O5^ii^	0.86	2.22	2.994 (4)	149
N2—H2*A*⋯O4^iii^	0.86	2.13	2.984 (4)	169
C7—H7*A*⋯O4^iv^	0.93	2.57	3.491 (4)	170
C9—H9*A*⋯*Cg*2^v^	0.98	2.83	3.737 (3)	154

Symmetry codes: (i) 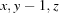; (ii) 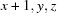; (iii) 
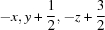
; (iv) 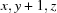; (v) 
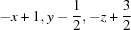
.

**Fig. 1.**
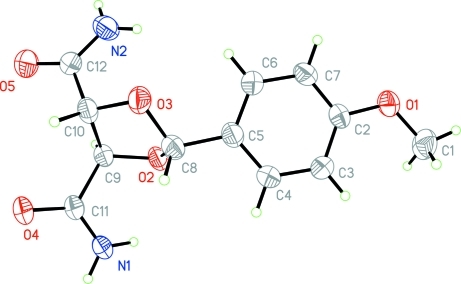
The molecular structure of the title compound, showing the atom-numbering and displacement ellipsoids drawn at the 30% probability level.

**Fig. 2.**
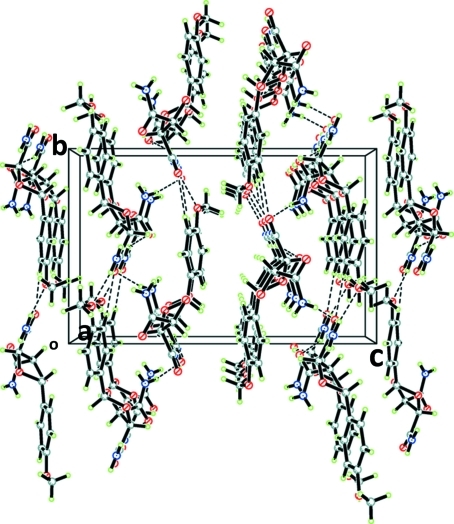
The crystal packing diagram of the title compound viewed along the a axis, with the N-H···O and C-H···O hydrogen bonds shown as dashed lines.

## supplementary crystallographic information

### Comment

Antitumor platinum drugs are some of the most effective anticancer agents currently available. (2*S*,3S)-Diethyl-2,3-*O*-alkyltartrate analogues are starting materials for the synthesis of platinum complexes with antitumor activity (Kim *et al.*, 1994), and are also important intermediates in organic synthesis (Pandey *et al.*, 1997). As part of our studies of the synthesis and characterization of such compounds, we herein report on the crystal structure of the title compound.

The molecular structure of the title compound is shown in Fig. 1. The bond lengths (Allen *et al.*, 1987) and angles are within normal ranges. The five-membered 1,3-dioxolane ring (O2,O3,C8-C10) has a twisted conformation on bond O2-C8.

In the crystal, intermolecular N—H···O and C—H···O hydrogen bonds link the molecules to form two-dimensional networks lieing parallel to the ab plane (Table 1 and Fig 2). There are also C-H···π interactions present in the crystal structure (Table 1).

### Experimental

The title compound was synthesized according to the published procedure (Ates-Alagoz & Buyukbingol, 2001). A mixture of (2*S*,3S)-diethyltartrate (500 mg, 2.43 mmol), 4-methoxybenzaldehyde (331 mg, 2.43 mmol), anhydrous copper(II) sulfate (776 mg, 2.86 mmol), and one drop of methane sulfonic acid in anhydrous toluene (8 ml) was stirred at room temperature for 8 h. Anhydrous Magnesium sulfate (30 mg) was added to the reaction mixture, which was then stirred for a further 20 min. Then a colourless precipitate was obtained by evaporation and dried in vacuo (Yield 83%). The obtained colourless product (654 mg, 2 mmol) was dissolved in 40 ml anhydrous ethanol, then a current of dry ammonia (dried by calcium cholride) was passed into the reaction mixture at room temperature for 4 h. The reaction mixture was then filtered and the resulting product was evaporated to dryness. Pure compound was obtained by crystallization from ethanol. Block-like yellow crystals of the title compound, suitable for X-ray diffraction, were obatined by slow evaporation of a solution in methanol after four weeks.

### Refinement

The NH and C-bound H-atoms were included in calculated positions and treated as riding atoms: N-H = 0.86 Å, C-H = 0.93, 0.98 and 0.96 Å for CH(aromatic), CH(methine), and CH_3_ H-atoms, respectively, with U_iso_(H) = k × U_eq_(C,N), where k = 1.5 for CH_3_ H-atoms, and k = 1.2 for all other H-atoms. In the final cycles of refinement, in the absence of significant anomalous scattering effects, 945 Friedel pairs were merged and Δf " set to zero.

### Crystal data

**Table:**

C_12_H_14_N_2_O_5_	*F*(000) = 560
*M**_r_* = 266.25	*D*_x_ = 1.399 Mg m^−^^3^
Orthorhombic, *P*2_1_2_1_2_1_	Mo *K*α radiation, λ = 0.71073 Å
Hall symbol: P 2ac 2ab	Cell parameters from 25 reflections
*a* = 6.9620 (14) Å	θ = 9–12°
*b* = 10.727 (2) Å	µ = 0.11 mm^−^^1^
*c* = 16.932 (3) Å	*T* = 293 K
*V* = 1264.5 (4) Å^3^	Block, yellow
*Z* = 4	0.20 × 0.20 × 0.10 mm

### Data collection

**Table:**

Enraf–Nonius CAD-4 diffractometer	1939 reflections with *I* > 2σ(*I*)
Radiation source: fine-focus sealed tube	*R*_int_ = 0.035
Graphite monochromator	θ_max_ = 25.3°, θ_min_ = 2.3°
ω/2θ scans	*h* = 0→8
Absorption correction: ψ scan (North *et al.*, 1968)	*k* = 0→12
*T*_min_ = 0.978, *T*_max_ = 0.989	*l* = −20→20
2615 measured reflections	3 standard reflections every 200 reflections
2297 independent reflections	intensity decay: 1%

### Refinement

**Table:**

Refinement on *F*^2^	Secondary atom site location: difference Fourier map
Least-squares matrix: full	Hydrogen site location: inferred from neighbouring sites
*R*[*F*^2^ > 2σ(*F*^2^)] = 0.041	H-atom parameters constrained
*wR*(*F*^2^) = 0.135	*w* = 1/[σ^2^(*F*_o_^2^) + (0.098*P*)^2^] where *P* = (*F*_o_^2^ + 2*F*_c_^2^)/3
*S* = 1.00	(Δ/σ)_max_ < 0.001
2297 reflections	Δρ_max_ = 0.16 e Å^−^^3^
173 parameters	Δρ_min_ = −0.16 e Å^−^^3^
0 restraints	Extinction correction: *SHELXL97* (Sheldrick, 2008), Fc^*^=kFc[1+0.001xFc^2^λ^3^/sin(2θ)]^-1/4^
Primary atom site location: structure-invariant direct methods	Extinction coefficient: 0.024 (5)

### Special details

**Table:**

Geometry. All e.s.d.'s (except the e.s.d. in the dihedral angle between two l.s. planes) are estimated using the full covariance matrix. The cell e.s.d.'s are taken into account individually in the estimation of e.s.d.'s in distances, angles and torsion angles; correlations between e.s.d.'s in cell parameters are only used when they are defined by crystal symmetry. An approximate (isotropic) treatment of cell e.s.d.'s is used for estimating e.s.d.'s involving l.s. planes.
Refinement. Refinement of *F*^2^ against ALL reflections. The weighted *R*-factor *wR* and goodness of fit *S* are based on *F*^2^, conventional *R*-factors *R* are based on *F*, with *F* set to zero for negative *F*^2^. The threshold expression of *F*^2^ > σ(*F*^2^) is used only for calculating *R*-factors(gt) *etc*. and is not relevant to the choice of reflections for refinement. *R*-factors based on *F*^2^ are statistically about twice as large as those based on *F*, and *R*- factors based on ALL data will be even larger.

### Fractional atomic coordinates and isotropic or equivalent isotropic displacement parameters (Å^2^)

**Table:**

	*x*	*y*	*z*	*U*_iso_*/*U*_eq_	
O1	0.7648 (3)	0.69904 (17)	0.91700 (13)	0.0480 (5)	
N1	0.6002 (4)	−0.0645 (2)	0.84147 (17)	0.0547 (7)	
H1A	0.6483	−0.1329	0.8591	0.066*	
H1B	0.6746	−0.0050	0.8266	0.066*	
C1	0.9569 (4)	0.7107 (3)	0.9441 (2)	0.0572 (8)	
H1C	0.9933	0.7970	0.9441	0.086*	
H1D	1.0410	0.6649	0.9098	0.086*	
H1E	0.9665	0.6781	0.9968	0.086*	
O2	0.4731 (3)	0.17224 (15)	0.81276 (11)	0.0394 (5)	
C2	0.6867 (4)	0.5817 (2)	0.91351 (15)	0.0369 (6)	
N2	−0.0220 (4)	0.2673 (2)	0.76177 (16)	0.0544 (7)	
H2A	−0.1036	0.2865	0.7256	0.065*	
H2B	0.0432	0.3249	0.7848	0.065*	
O3	0.2168 (3)	0.23134 (17)	0.88371 (11)	0.0417 (5)	
C3	0.7876 (4)	0.4738 (3)	0.93091 (17)	0.0436 (7)	
H3A	0.9146	0.4776	0.9477	0.052*	
O4	0.2966 (3)	−0.13078 (18)	0.85712 (14)	0.0524 (6)	
C4	0.6951 (4)	0.3598 (3)	0.92277 (17)	0.0440 (7)	
H4A	0.7617	0.2870	0.9348	0.053*	
O5	−0.0843 (3)	0.0617 (2)	0.75178 (15)	0.0587 (6)	
C5	0.5076 (4)	0.3515 (2)	0.89740 (15)	0.0383 (6)	
C6	0.4100 (4)	0.4611 (3)	0.88108 (18)	0.0439 (7)	
H6A	0.2829	0.4574	0.8643	0.053*	
C7	0.4980 (4)	0.5755 (3)	0.88927 (17)	0.0449 (6)	
H7A	0.4302	0.6482	0.8785	0.054*	
C8	0.4199 (4)	0.2252 (3)	0.88635 (16)	0.0394 (6)	
H8A	0.4601	0.1701	0.9294	0.047*	
C9	0.3416 (4)	0.0725 (2)	0.80307 (15)	0.0358 (6)	
H9A	0.3170	0.0612	0.7465	0.043*	
C10	0.1557 (4)	0.1216 (2)	0.84279 (16)	0.0389 (6)	
H10A	0.1077	0.0600	0.8807	0.047*	
C11	0.4124 (4)	−0.0509 (2)	0.83725 (17)	0.0404 (7)	
C12	0.0032 (4)	0.1493 (2)	0.78190 (16)	0.0413 (6)	

### Atomic displacement parameters (Å^2^)

**Table:**

	*U*^11^	*U*^22^	*U*^33^	*U*^12^	*U*^13^	*U*^23^
O1	0.0442 (11)	0.0306 (10)	0.0693 (13)	−0.0077 (9)	−0.0037 (9)	−0.0019 (9)
N1	0.0415 (14)	0.0295 (12)	0.093 (2)	−0.0034 (11)	−0.0067 (13)	0.0148 (14)
C1	0.0528 (19)	0.0486 (17)	0.070 (2)	−0.0190 (15)	−0.0076 (16)	0.0023 (16)
O2	0.0413 (11)	0.0243 (9)	0.0527 (10)	−0.0038 (8)	0.0054 (8)	0.0005 (8)
C2	0.0417 (15)	0.0281 (12)	0.0409 (13)	−0.0032 (12)	0.0013 (12)	−0.0011 (11)
N2	0.0564 (16)	0.0378 (13)	0.0689 (16)	0.0055 (12)	−0.0172 (14)	−0.0031 (12)
O3	0.0378 (10)	0.0356 (10)	0.0516 (11)	−0.0051 (8)	0.0018 (8)	−0.0084 (8)
C3	0.0383 (15)	0.0389 (15)	0.0537 (16)	−0.0010 (13)	−0.0111 (13)	0.0007 (12)
O4	0.0461 (12)	0.0288 (10)	0.0824 (14)	−0.0100 (9)	−0.0044 (11)	0.0089 (10)
C4	0.0453 (16)	0.0313 (14)	0.0554 (16)	0.0033 (13)	−0.0119 (13)	0.0029 (12)
O5	0.0483 (11)	0.0446 (12)	0.0833 (16)	−0.0088 (11)	−0.0161 (11)	−0.0066 (11)
C5	0.0448 (15)	0.0303 (12)	0.0398 (13)	−0.0030 (13)	−0.0042 (12)	0.0002 (10)
C6	0.0374 (14)	0.0341 (14)	0.0601 (17)	−0.0025 (12)	−0.0069 (13)	−0.0002 (12)
C7	0.0414 (15)	0.0285 (12)	0.0647 (17)	0.0025 (13)	−0.0014 (15)	0.0017 (12)
C8	0.0439 (15)	0.0314 (13)	0.0428 (14)	−0.0021 (12)	−0.0029 (12)	0.0058 (12)
C9	0.0380 (13)	0.0281 (12)	0.0413 (13)	−0.0038 (12)	−0.0020 (12)	0.0024 (11)
C10	0.0408 (15)	0.0274 (13)	0.0484 (14)	−0.0065 (11)	0.0033 (13)	0.0015 (11)
C11	0.0412 (15)	0.0261 (13)	0.0541 (16)	−0.0052 (12)	−0.0042 (13)	−0.0018 (11)
C12	0.0329 (12)	0.0352 (14)	0.0557 (16)	−0.0010 (13)	0.0008 (13)	−0.0032 (12)

### Geometric parameters (Å, º)

**Table:**

O1—C2	1.373 (3)	C3—C4	1.388 (4)
O1—C1	1.420 (4)	C3—H3A	0.9300
N1—C11	1.317 (4)	O4—C11	1.224 (3)
N1—H1A	0.8600	C4—C5	1.378 (4)
N1—H1B	0.8600	C4—H4A	0.9300
C1—H1C	0.9600	O5—C12	1.230 (3)
C1—H1D	0.9600	C5—C6	1.386 (4)
C1—H1E	0.9600	C5—C8	1.498 (4)
O2—C9	1.417 (3)	C6—C7	1.378 (4)
O2—C8	1.419 (3)	C6—H6A	0.9300
C2—C7	1.378 (4)	C7—H7A	0.9300
C2—C3	1.386 (4)	C8—H8A	0.9800
N2—C12	1.323 (4)	C9—C11	1.527 (4)
N2—H2A	0.8600	C9—C10	1.551 (4)
N2—H2B	0.8600	C9—H9A	0.9800
O3—C8	1.416 (3)	C10—C12	1.509 (4)
O3—C10	1.430 (3)	C10—H10A	0.9800
			
C2—O1—C1	117.9 (2)	C5—C6—H6A	119.4
C11—N1—H1A	120.0	C6—C7—C2	119.8 (3)
C11—N1—H1B	120.0	C6—C7—H7A	120.1
H1A—N1—H1B	120.0	C2—C7—H7A	120.1
O1—C1—H1C	109.5	O3—C8—O2	104.6 (2)
O1—C1—H1D	109.5	O3—C8—C5	111.7 (2)
H1C—C1—H1D	109.5	O2—C8—C5	111.4 (2)
O1—C1—H1E	109.5	O3—C8—H8A	109.7
H1C—C1—H1E	109.5	O2—C8—H8A	109.7
H1D—C1—H1E	109.5	C5—C8—H8A	109.7
C9—O2—C8	103.63 (19)	O2—C9—C11	113.7 (2)
O1—C2—C7	115.7 (2)	O2—C9—C10	103.43 (19)
O1—C2—C3	123.8 (2)	C11—C9—C10	113.6 (2)
C7—C2—C3	120.4 (3)	O2—C9—H9A	108.6
C12—N2—H2A	120.0	C11—C9—H9A	108.6
C12—N2—H2B	120.0	C10—C9—H9A	108.6
H2A—N2—H2B	120.0	O3—C10—C12	112.2 (2)
C8—O3—C10	105.9 (2)	O3—C10—C9	104.0 (2)
C2—C3—C4	118.6 (3)	C12—C10—C9	110.9 (2)
C2—C3—H3A	120.7	O3—C10—H10A	109.9
C4—C3—H3A	120.7	C12—C10—H10A	109.9
C5—C4—C3	121.8 (3)	C9—C10—H10A	109.9
C5—C4—H4A	119.1	O4—C11—N1	124.1 (3)
C3—C4—H4A	119.1	O4—C11—C9	119.9 (2)
C4—C5—C6	118.2 (3)	N1—C11—C9	115.9 (2)
C4—C5—C8	118.9 (2)	O5—C12—N2	123.9 (3)
C6—C5—C8	122.9 (2)	O5—C12—C10	118.8 (2)
C7—C6—C5	121.2 (3)	N2—C12—C10	117.2 (2)
C7—C6—H6A	119.4		
			
C1—O1—C2—C7	178.0 (3)	C4—C5—C8—O2	−81.2 (3)
C1—O1—C2—C3	−3.5 (4)	C6—C5—C8—O2	96.9 (3)
O1—C2—C3—C4	−177.9 (3)	C8—O2—C9—C11	−90.3 (3)
C7—C2—C3—C4	0.5 (4)	C8—O2—C9—C10	33.4 (2)
C2—C3—C4—C5	0.5 (4)	C8—O3—C10—C12	−135.0 (2)
C3—C4—C5—C6	−1.1 (4)	C8—O3—C10—C9	−15.0 (3)
C3—C4—C5—C8	177.2 (3)	O2—C9—C10—O3	−11.4 (3)
C4—C5—C6—C7	0.5 (4)	C11—C9—C10—O3	112.3 (2)
C8—C5—C6—C7	−177.6 (3)	O2—C9—C10—C12	109.4 (2)
C5—C6—C7—C2	0.5 (4)	C11—C9—C10—C12	−126.9 (2)
O1—C2—C7—C6	177.5 (3)	O2—C9—C11—O4	155.4 (3)
C3—C2—C7—C6	−1.0 (4)	C10—C9—C11—O4	37.5 (4)
C10—O3—C8—O2	36.7 (3)	O2—C9—C11—N1	−26.2 (4)
C10—O3—C8—C5	157.3 (2)	C10—C9—C11—N1	−144.1 (3)
C9—O2—C8—O3	−44.4 (2)	O3—C10—C12—O5	−168.2 (3)
C9—O2—C8—C5	−165.1 (2)	C9—C10—C12—O5	76.0 (3)
C4—C5—C8—O3	162.3 (2)	O3—C10—C12—N2	14.7 (4)
C6—C5—C8—O3	−19.6 (4)	C9—C10—C12—N2	−101.1 (3)

### Hydrogen-bond geometry (Å, º)

Cg2 is the centroid of the C2–C7 ring.

**Table:**

*D*—H···*A*	*D*—H	H···*A*	*D*···*A*	*D*—H···*A*
N1—H1*A*···O1^i^	0.86	2.21	3.063 (3)	174
N1—H1*B*···O5^ii^	0.86	2.22	2.994 (4)	149
N2—H2*A*···O4^iii^	0.86	2.13	2.984 (4)	169
C7—H7*A*···O4^iv^	0.93	2.57	3.491 (4)	170
C9—H9*A*···*Cg*2^v^	0.98	2.83	3.737 (3)	154

Symmetry codes: (i) *x*, *y*−1, *z*; (ii) *x*+1, *y*, *z*; (iii) −*x*, *y*+1/2, −*z*+3/2; (iv) *x*, *y*+1, *z*; (v) −*x*+1, *y*−1/2, −*z*+3/2.
